# Targeting the Notch and TGF-β signaling pathways to prevent retinal fibrosis *in vitro* and *in vivo*

**DOI:** 10.7150/thno.45192

**Published:** 2020-06-29

**Authors:** Jiawen Fan, Weiyong Shen, So-Ra Lee, Ashish Easow Mathai, Rui Zhang, Gezhi Xu, Mark C. Gillies

**Affiliations:** 1 The University of Sydney, Save Sight Institute, Discipline of Ophthalmology, Sydney Medical School, Sydney, New South Wales, Australia.; 2 Department of Ophthalmology and Vision Sciences and Key Laboratory of Myopia of State Health Ministry, Eye and ENT Hospital, Shanghai Medical College, Fudan University, Shanghai, People's Republic of China.

**Keywords:** Notch, transforming growth factor β, signalling pathway, Müller cells, retina, fibrosis

## Abstract

**Rationale:** The Notch and transforming growth factor-β (TGFβ) signaling pathways are two intracellular mechanisms that control fibrosis in general but whether they play a major role in retinal fibrosis is less clear. Here we study how these two signaling pathways regulate Müller cell-dominated retinal fibrosis *in vitro* and* in vivo*.

**Methods:** Human MIO-M1 Müller cells were treated with Notch ligands and TGFβ1, either alone or in combination. Western blots were performed to study changes in γ-secretase proteases, Notch downstream effectors, endogenous TGFβ1, phosphorylated Smad3 (p-Smad3) and extracellular matrix (ECM) proteins. We also studied the effects of RO4929097, a selective γ-secretase inhibitor, on expression of ECM proteins after ligand stimulation. Müller cell viability was studied by AlamarBlue and cytotoxicity by lactate cytotoxicity assays. Finally, we studied changes in Notch and TGFβ signaling and tested the effect of intravitreal injections of the Notch pathway inhibitor RO4929097 on retinal fibrosis resulted from Sodium iodate (NaIO_3_)-induced retinal injury in mice. We also studied the safety of intravitreal injections of RO4929097 in normal mice.

**Results:** Treatment of Müller cells with Notch ligands upregulated γ-secretase proteases and Notch downstream effectors, with increased expression of endogenous TGFβ1, TGFβ receptors and p-Smad3. TGFβ1 upregulated the expression of proteins associated with both signaling pathways in a similar manner. Notch ligands and TGFβ1 had additive effects on overexpression of ECM proteins in Müller cells which were inhibited by RO4929097. Notch and TGFβ ligands stimulated Müller cell proliferation which was inhibited by RO4929097 without damaging the cells. NaIO_3_-induced retinal injury activated both Notch and TGFβ signaling pathways *in vivo*. Intravitreal injection of RO4929097 prevented Müller cell gliosis and inhibited overexpression of ECM proteins in this murine model. We found no safety concerns for up to 17 days after an intravitreal injection of RO4929097.

**Conclusions:** Inhibiting Notch signaling might be an effective way to prevent retinal fibrosis. This study is of clinical significance in developing a treatment for preventing fibrosis in proliferative vitreoretinopathy, proliferative diabetic retinopathy and wet age-related macular degeneration.

## Introduction

Normal vision relies on a clear visual axis, well-organised retinal architecture and normally functioning cellular compartments for phototransduction. Retinal fibrosis (scarring) develops in response to acute or chronic retinal injury including inflammation, ischemia and neurodegeneration 1-4. Fibrosis alters the retinal architecture and disrupts the normal cell-cell relationships, resulting in impaired vision. Retinal fibrosis is irreversible once it has become established and currently there is no treatment to either prevent it or treat it.

Retinal fibrosis occurs in many retinal diseases including proliferative vitreoretinopathy (PVR) 1-3, proliferative diabetic retinopathy (PDR) 2, 4, neovascular (“wet') age-related macular degeneration (nAMD) 5-7 and inherited retinal degenerations (IRDs) 8. Uncontrolled proliferation of fibrotic tissue in proliferative retinopathy results in traction retinal detachment.

Subretinal fibrosis has been identified as a major cause of poor outcomes for anti-VEGF therapy in nAMD which is characterized by the growth of abnormal vessels under the retina 5-7. These vessels may respond to intravitreal injections of vascular endothelial growth factor (VEGF) inhibitors but vision may still be lost due to the development of subretinal fibrosis. Approximately half of eyes with nAMD develop it within 2 years of starting anti-VEGF therapy 7, 9. Retinal fibrosis also occurs in IRDs secondary to retinal degeneration due to genetic mutations 8. Recent advance in stem cell biology has made cell replacement therapy potentially feasible for IRDs. However, the formation of fibrotic tissue between the host neuroretina and the grafted cells/tissue has been implicated as one of the reasons for failure of subretinal cell replacement therapy to improve vision in IRDs 10.

Retinal fibrosis is characterized by excessive deposition of extracellular matrix (ECM) proteins by activated Müller cells, astrocytes, microglia, transformed retinal pigment epithelium (RPE) cells, myofibroblast-like cells and vascular endothelial cells 5, 6, 11. Müller glial cells and RPE cells play important roles in the maintenance of normal retinal homeostasis. In diseased conditions, however, Müller cells become gliotic and RPE cells differentiate into myofibroblast-like cells through epithelial-mesenchymal transition (EMT). It has been well documented that gliotic Müller cells and transdifferentiated RPE cells contribute to retinal fibrosis in PVR, PDR, nAMD and IRDs 5, 8, 11-13.

The Notch signaling pathway is an important intracellular mechanism 14-17 that controls fibrosis in other organs 18, 19. Ligand-receptor interaction leads to the cleavage of the Notch intracellular domain (NICD) by γ-secretase proteases, followed by its translocation to the nucleus where the cleaved NICD regulates the expression of downstream target genes including those that contribute to fibrosis 20-22. γ-secretase is a tetrameric complex formed by nicastrin, anterior pharynx defective 1, presenilin enhancer 2 (PEN2) and presenilin. Stoichiometric assembly of these subunits is required to form a functional complex and disruption of this complex will result in its rapid degradation 23. Inhibition of NICD cleavage using a γ-secretase inhibitor has been reported to reduce expression of Notch transcriptional target genes in cancer research 24, 25. Notch signaling regulates Müller cell differentiation and proliferation 26, 27. Notch signaling has been reported to promote TGF-β1-induced EMT of RPE cells and contribute to fibrosis in animal models of PVR, both of which can be inhibited by γ-secretase inhibitors including LY411575 and DAPT 28, 29. These observations indicate that Notch signaling is an important intracellular mechanism in RPE-mediated retinal fibrosis.

The transforming growth factor β (TGFβ) signaling pathway is also pro-fibrotic 5, 6, 30 and could be the target of anti-fibrotic therapy 31, 32. Interaction of TGFβ receptors with their ligands results in phosphorylation of Smad proteins, leading to nuclear translocation and regulation of pro-fibrotic gene expression. TGFβ signaling regulates the production of ECM proteins and is involved in migration and proliferation of fibroblasts 33-35 and vascular endothelial cells 36-38. It also promotes EMT of the RPE 5, 6, 30. We have previously reported that TGFβ signaling is activated during Müller cell gliosis in transgenic mice with induced Müller cell disruption 39. We found that intravitreal injection of an antibody against endoglin, a co-receptor essential for TGFβ signaling, attenuated protrusion of Müller cell processes into the subretinal space resuling from induced Müller cell disruption 39. These collective observations indicate that TGFβ signaling also plays an important role in EMT of RPE cells and Müller cell gliosis.

The contribution of Notch and TGFβ signaling to EMT of RPE cells and subsequent development of RPE-mediated retinal fibrosis is generally well studied *in vitro* 29, 40-42 and *in vivo* 43-45 but how these two signaling pathways regulate Müller cell-dominated retinal fibrosis is less clear. Here, we studied whether the Notch and TGFβ signaling pathways contribute to retinal fibrosis using human Müller cells as an *in vitro* model. We also particularly studied the effect of RO4929097, a selective γ-secretase inhibitor which has a much stronger potency in inhibiting Notch signaling than DAPT 46, on retinal fibrosis *in vitro* and *in vivo*.

## Methods

### Culture of MIO-M1 human Müller cells

MIO-M1 Müller cells (RRID: CVCL_0433) were cultured in DMEM media (Invitrogen#11885) containing 4.5 g/L D-glucose, 110 mg/L sodium pyruvate, 1% penicillin/streptomycin and 10% fetal bovine serum (FBS) unless specified. We used Nunc™ EasYFlask™ Cell Culture Flasks (Cat# 156367) and Nunc™ Non-Treated Multidishes (Cat# 150200) to culture MIO-M1 Müller cells. In order to optimize the time points of Notch and TGFβ ligand treatment for activation of these two signaling pathways, Müller cells were cultured in DMEM media until 80% confluence and then incubated in media containing recombinant human Notch ligands including Delta-like 4 (Dll4, R&D Systems, Cat#1506-D4, 50 ng/ml) and Jagged-1 (Jag1, R&D Systems, Cat# 277-JG, 50 ng/ml) or recombinant human TGFβ1 (R&D Systems, Cat#240-B, 10 ng/ml) for 3, 6, 18 and 24 hours. Changes in Notch and TGFβ signaling proteins were studied by Western Blots using antibodies against γ-secretase proteases, Notch downstream effectors, total and phosphorylated Smad 2 and 3 (**Table 1**).

In order to study the effects of Notch inhibition on the events associated with retinal fibrosis, we also incubated Müller cells in media containing Notch ligands including Dll4 and Jag1 (both 50 ng/ml) and TGFβ1 (10 ng/ml) with or without 1 or 10 µM of the γ-secretase protease inhibitor RO4929097 (Selleckchem, Cat#S1575, stock solution 50 mM dissolved in DMSO). Changes in Notch and TGFβ signaling and ECM proteins were studied by Western blots (**Table 1**).

### AlamarBlue cell viability assay

We performed AlamarBlue cell viability assays to test the effects of ligand treatment on Müller cell proliferation. Briefly, Müller cells were seeded at a density of 1×10^4^ cells/well in 96-well plates (Corning) and cultured until 80% confluence. After starving cells in serum-free DEME media containing 1% penicillin-streptomycin and 1% insulin-transferrin-selenium-ethanolamine supplements (ITS-X, GIOCO#51500056) overnight, MIO-M1 cells were incubated in media containing 1% FBS, 1% penicillin-streptomycin, 1% ITS and test compounds for 24 hours. Cell viability was assessed by adding 10 μl of AlamarBlue (Invitrogen) to 100 μl of media in each well followed by one-hour incubation in a CO_2_ incubator. The resulting fluorescence was read with a Tecan Safire2 fluorescence multi-well plate reader (Tecan, Switzerland) as described previously.

### Lactate dehydrogenase (LDH) cytotoxicity assay

We performed LDH cytotoxicity assays (Pierce, Cat# 88953) to study the potential damage of Notch inhibition to Müller cells. Briefly, 50 μl of culture medium was collected from each well after treating Müller cells with Notch and TGFβ ligands, either with or without the presence of 10 μM of RO4929097. After centrifugation, 15 μl of supernatants from each respective group was added into each well of a 384-well plate followed by mixing with 15 μl of LDH reaction reagent. After incubating the mixed solution at room temperature for 30 minutes, reactions were read using TECAN plate reader with the absorbance at 490 nm and 680 nm. LDH activity was determined by subtracting the reading at 680 nm absorbance from the value at 490 nm absorbance. All samples were run with eight biological replicates and two technical repeats.

### Animals

The animal studies were approved by The University of Sydney Animal Ethics Committee and performed in accordance with the Association of Ophthalmology and Vision Research (ARVO) statement for the use of animals in Ophthalmology and Vision Research. Male and female C57BL/6J mice aged 3-4 months of age were housed in a temperature and humidity-controlled rooms with a 12-hour light and 12-hour dark cycle and provided with food and water ad libitum. Seventy-five mice were used for Sodium iodate (NaIO_3_)-induced model and 22 mice were used to study the safety of an intravitreal injection of RO4929097 in the normal retina as described below.

### Intravitreal injections in mice with NaIO_3_-induced retinal degeneration

Intravitreal injections of test agents were performed using a 32-gauge needle attached to a Hamilton syringe as described previously 47-49. Briefly, mice were anaesthetized with ketamine (48 mg/kg) and medetomidine (0.6 mg/kg) and their pupils dilated with 1-2 drops of 1% tropicamide and 0.5% phenylephrine. The conjunctiva was cut close to the limbus and the sclera was exposed. A shelving puncture (45-degree angle) of the sclera was made 2-3 mm behand the limbus with a 30-gauge needle. The 32-gauge Hamilton needle was introduced through this hole under an operating microscope and 1.0 µl of 100 μM RO4929097 was delivered into the vitreous cavity, with the contralateral eye injected with phosphate-buffered saline (PBS) containing 0.2% DMSO as a control. A fine forceps was used to block liquid reflux by pressing the rear site of the shelving puncture when the Hamilton needle was withdrawn from the vitreous. Immediately after intravitreal injection, mice received one dose of intraperitoneal injection of NaIO_3_ (50 mg/kg, Sigma-Aldrich Corp., St. Louis, MO, USA) to induce damage to the RPE. Eyes were enucleated 5 days after treatment to study changes in Notch and TGFβ signaling, Müller cell gliosis and photoreceptor degeneration.

### Safety study on intravitreal injection of RO4929097 in the normal retina

In order to study the safety of intravitreal injection of RO4929097, some normal mice received 1.0 μl of RO4929097, PBS, PBS containing 0.2% DMSO or no injection and the injected mice were examined by color fundus photography and fluorescein angiography one week after injection. Eyes were also enucleated 10 days after angiography for cone arrestin (Millipore #AB15282, 1:500) immunostaining in retinal wholemounts to study changes in photoreceptors as described below.

### Immunohistochemistry (IHC) in frozen sections and retinal wholemounts

Eyes were fixed in 4% paraformaldehyde for one hour, transferred to PBS containing 30% sucrose for 2-3 hours and then embedded in optimal cutting temperature compound (Tissue-Tek; Sakura Finetek, Torrance, CA) as described previously 47-49. Frozen sections were incubated with antibodies against glial fibrillary acidic protein (GFAP, Dako#Z0334, 1:250) to study Müller cell gliosis and RPE65 (Novus#NB-100-355, 1:200) to identify RPE cells. CRALBP (Abcam#15051, 1:200) were used for phenotypic characterisation of Müller cells. Alpha smooth muscle actin (α-SMA, Cell Signalling#48938, 1:200) and Ionized calcium binding adaptor molecule 1 (Iba1, Wako#019-19741, 1:500) were used to identify differentiated fibroblasts and microglias. We used peanut-agglutinin (PNA) conjugated with Alexa Fluor 594 (10 µg/ml, Invitrogen, L-32459) to study changes in cone photoreceptor apical processes. After incubating with primary antibodies at +4°C overnight, the bound antibodies were detected with corresponding secondary antibodies conjugated with Alexa Fluor 488 or 594 (1:1000; Invitrogen) and then examined by confocal laser scanning microscopy.

For immunostaining in retinal wholemounts, dissected eye cups were fixed in 4% paraformaldehyde for one hour and then placed in PBS at +4°C. On next day, retinas were isolated, permeabilized and incubated in a solution containing PNA conjugated with Alexa Fluor 594 (10 µg/ml, Invitrogen, L-32459) or an antibody against cone arrestin (Millipore #AB15282, 1:500) to study changes in cone photoreceptor apical processes. Changes in preretinal and subretinal Müller cell gliosis was studied using an antibody against GFAP (Dako#Z0334, 1:250). Iba1 (Wako#019-19741, 1:500) was applied to study microglial activation. Retinal wholemounts were counterstained with Hoechst stain and confocal images were processed and quantitatively analysed as previously described 47-49. In brief, images were processed and analysed using computer-based image analysis software (Image J) to determine the percentage of PNA, cone arrestin, GFAP or Iba1 stained area per field of view as described previously 47, 48. A gradient detection algorithm was applied to the original digital image and binary thresholding performed on the gradient image by selecting its mean gray value as the threshold. This procedure allowed sufficient identification of the subject profiles to calculate the percentage of PNA, cone arrestin, GFAP or Iba1-stained area per field of view.

### Western blot analysis

Western blots were conducted to study changes in Notch and TGFβ signaling, expression of ECM proteins and Müller cell gliosis as described previously 50. Treated MIO-M1 cells and retina tissues were lysed using RIPA buffer (Sigma Aldrich) with protease and phosphatase inhibitors (Roche). Protein concentrations were determined by bicinchoninic acid assays (QuantiPro™ BCA Assay Kit, Sigma Aldrich, Cat# QPBCA). Lysate proteins were separated on NuPAGE Novex 4-12% Bis-Tris 1 mm-thick mini-gels (Life Technologies, USA) by electrophoresis and then transferred to polyvinylidene fluoride membranes (PVDF, Millipore) for blotting. The membranes were probed with primary Abs overnight at 4 °C (**Table 1**) and then incubated with secondary Abs conjugated with horseradish peroxidise at room temperature for 2 hours. Protein bands were visualised using the G:Box BioImaging system and quantified using the GeneTools image scanning and analysis package. Protein expression was normalised to β-actin (Cell Signaling#4967, 1:2000) in *in vitro* and to α/β-tubulin (rabbit polyclonal, 1:2000; Cell Signaling #2148) in *in vivo* studies, both of which served as loading controls. The value of normalised densitometry was compared as a ratio of control in each group.

### Statistical analysis

Data were shown as mean ± standard error of mean (SEM). GraphPad Prism 7.0 and SPSS 17.0 for Windows were used for statistical analysis. Comparisons between two groups were made using paired or unpaired t-test. Statistical evaluation among multiple groups was conducted by one-way ANOVA followed by Tukey's Multiple Comparison Test (Homogeneous Variances). A p value <0.05 was regarded as statistically significant.

## Results

### Activation of the Notch and TGFβ signaling pathways in human MIO-M1 Müller cells treated with Notch and TGFβ ligands

**Figure 1:**
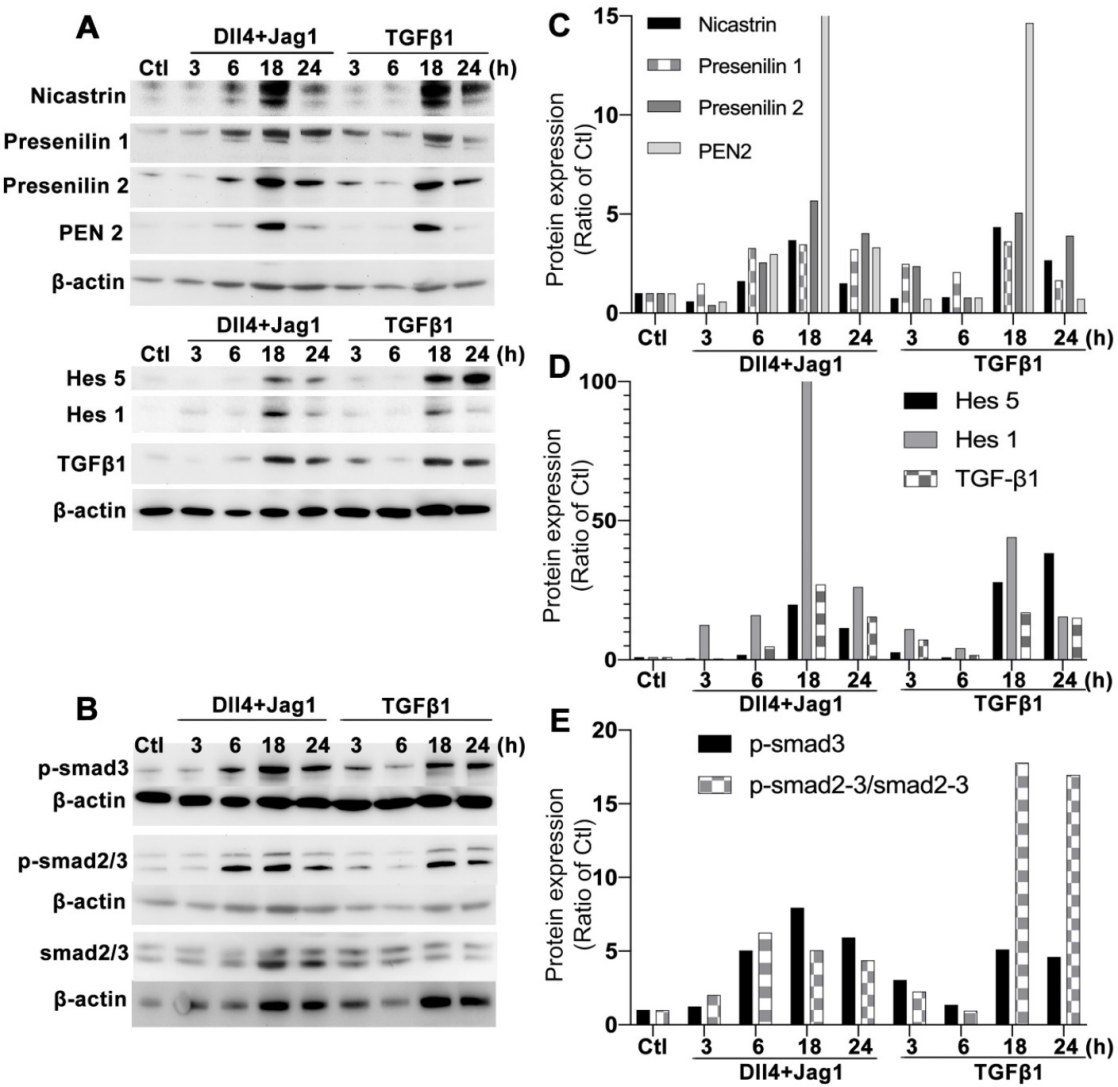
** Optimising timepoints for activation of Notch and TGFβ signalling pathways in MIO-M1 human Müller cells.** Western blots were conducted after culturing Müller cells in normal (control, Ctl) and test media containing recombinant human Notch ligands including Dll4 and Jagged 1 (Jag1, both 50 ng/ml) or TGFβ1 (10 ng/ml) for 3, 6 18 and 24 hours. **(A)** Changes in γ-secretase proteinases including nicastrin, presenilin 1 and 2, presenilin enhancer 2 (PEN2) and Notch downstream effectors including endogenous TGFβ1, Hes1 and Hes5. **(B)** Changes in total and phosphorylated Smad 2/3 (p-Smad 2/3). Treatment of Müller cells with Notch or TGFβ ligands for 18 or 24 hours induced extensive activation of both Notch and TGFβ signalling pathways. Independent repeats=2. **(C-E)** Densitometry measurements of proteins in the Notch and TGF signaling pathways after normalization to the housekeeping protein β-actin.

We performed Western blots to study changes in the Notch and TGFβ signaling pathways after treating Müller cells with Notch ligands or TGFβ1. Treatment of Müller cells with Dll4 and Jagged1 or TGFβ1 upregulated γ-secretase proteases including presenilin 1 and 2, nicastrin and PEN2 as early as 6 hours after treatment, with strong expression observed 18 hours after treatment (Figure 1A). Similar changes were observed in Notch downstream effectors including hairy and enhancer of split-1 (Hes1) and Hes5 (Figure 1A). Treatment of Müller cells with Notch ligands or TGFβ1 also resulted in increased production of endogenous TGFβ1 (Figure 1A) and upregulation of p-Smad3 (Figure 1B). Normalization of the densitometry of each protein band to the housekeeping protein β-actin indicated that treatment with either Notch ligands or TGFβ1 for 18 or 24 hours profoundly activated each signaling pathway in Müller cells (Figure 1C-E).

### RO4929097 inhibited both Notch and TGFβ signaling pathways in Müller cells stimulated by Notch ligands

**Figure 2:**
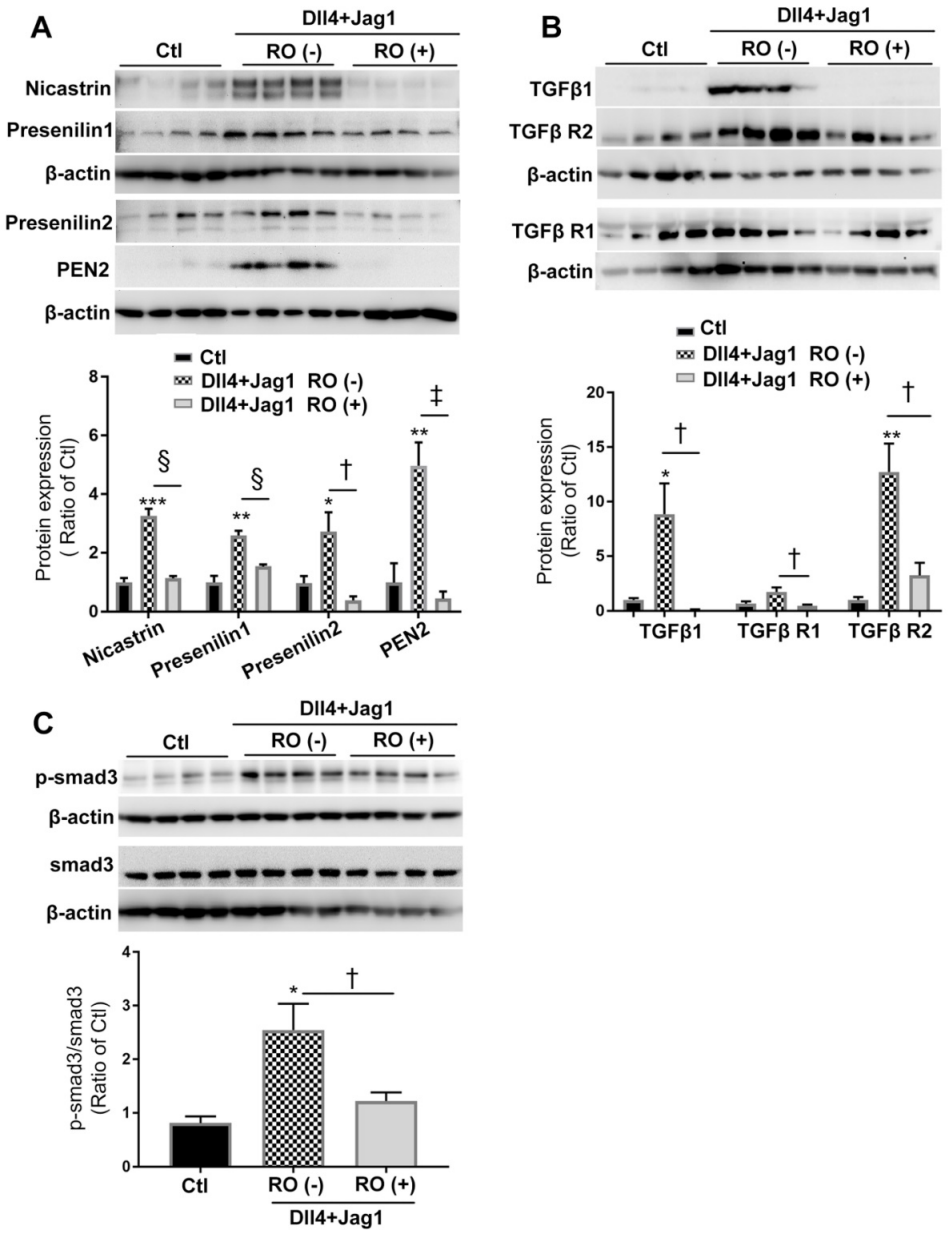
** RO4929097 (RO) inhibits Notch and TGFβ signalling in Müller cells stimulated by Notch ligands.** MIO-M1 human Müller cells were cultured in normal (control, Ctl) and test media containing Notch ligands including Dll4 and Jagged1 (both 50 ng/ml), either with or without RO (10 μM) for 18 hours. **(A)** Treatment of Müller cells with Dll4 and Jag1 upregulated the expression of γ-secretase proteinases including nicastrin, presenilin 1 and 2 as well as presenilin enhancer 2 (PEN2), all of which were significantly inhibited by the selective γ-secretase inhibitor RO. **(B and C)** Treatment of Müller cells with Dll4 and Jag1 also upregulated the expression of TGFβ1, TGFβ receptors 1 and 2 (TGFβ R1 and TGFβ R2, (**B**) and p-Smad3 (**C**), all of which were significantly inhibited by RO treatment. *P<0.05, **P<0.01 and ***P<0.001, vs control (Ctl). ^†^P<0.05, ^‡^P<0.01 and ^§^P<0.001 vs the corresponding groups without RO treatment. N=4/group. Independent repeats=2.

We next studied the effect of RO4929097, a selective γ-secretase protease inhibitor, on Notch and TGFβ signaling in Müller cells treated with Notch ligands for 18 hours (Figure 2). Consistent with our observations shown in Figure 1, Dll4 and Jagged1 significantly upregulated the expression of γ-secretase proteinases including nicastrin, presenilin 1 and 2 and PEN2 (Figure 2A), accompanied by increased expression of endogenous TGFβ1, TGFβ receptor type 1 and type 2 receptors (TGFβ-R1 and TGFβ-R2, Figure 2B) and p-Smad3 (Figure 2C) and these changes were significantly inhibited by RO4929097 (Figure 2B-C). These results indicate that RO4929097 inhibits the activation of both signaling pathways resulting from treatment with ligands for either signaling pathway.

### RO4929097 also inhibited both Notch and TGFβ signaling pathways in Müller cells stimulated by TGFβ1

**Figure 3:**
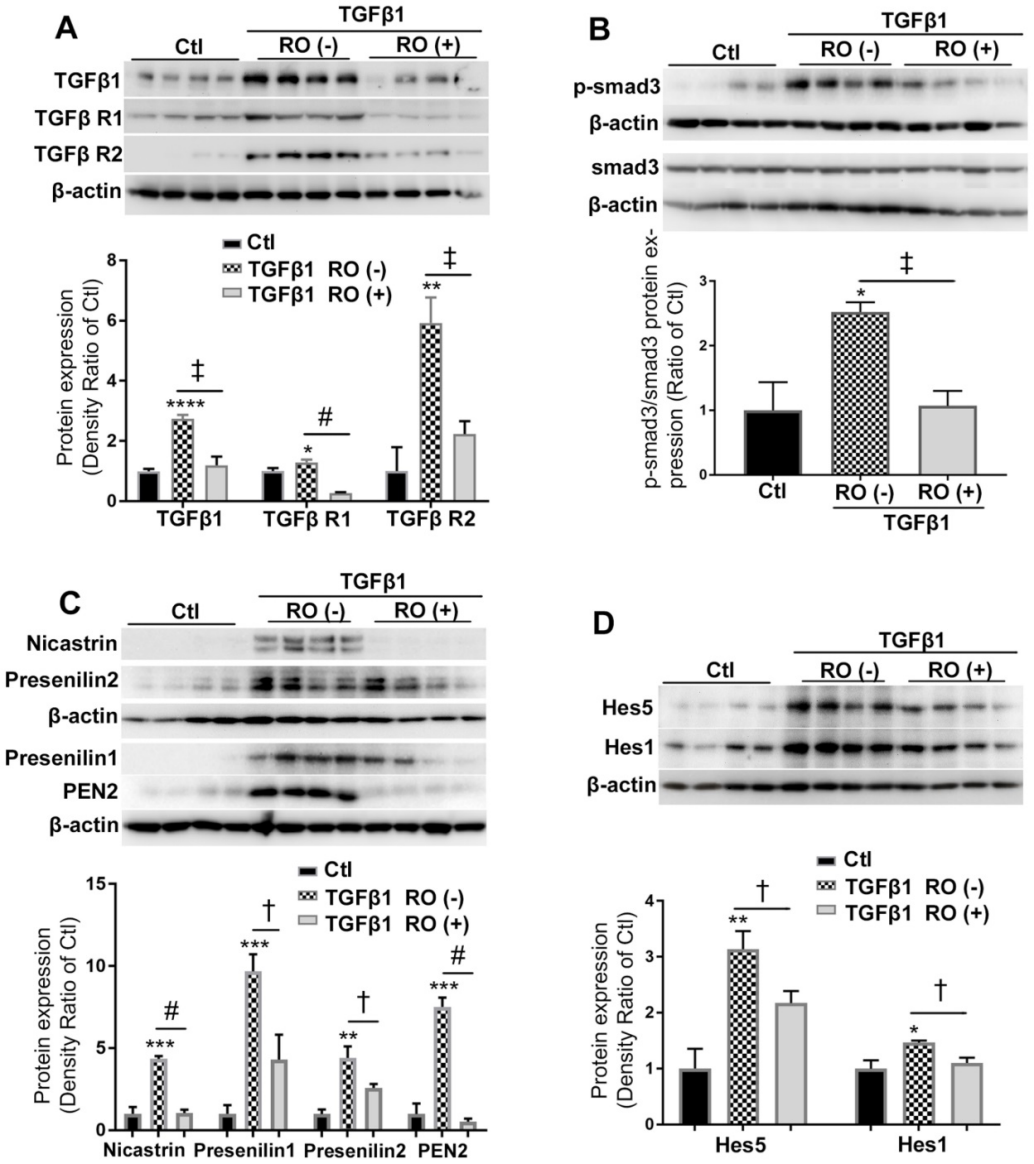
** RO4929097 (RO) inhibits TGFβ and Notch signalling in Müller cells stimulated by TGFβ1.** Müller cells were cultured in normal (control, Ctl) and test media supplemented with recombinant human TGFβ1 (10 ng/ml), with or without RO (10 μM) for 18 hours. **(A and B)** TGFβ1 treatment upregulated expression of TGFβ receptors including TGFβ R1 and 2, endogenous TGFβ1 (A) and p-Smad3 (B) while this effect was significantly inhibited by RO. **(C and D)** TGFβ1 also significantly upregulated expression of γ-secretase proteinases including nicastrin, presenilin 1 and 2, presenilin enhancer 2 (PEN2) as well as Notch downstream effectors including Hes1 and Hes5, while this effect was inhibited by RO treatment. *P<0.05, **P<0.01 and ***P<0.001, vs control (Ctl). ^†^P<0.05, ^‡^P<0.01 and ^#^P<0.0001 vs the corresponding groups without RO treatment. N=4/group. Independent repeats=2.

We also studied the effect of RO4929097 on TGFβ and Notch signaling in Müller cells treated with TGFβ1 (Figure 3). Stimulation of Müller cells with TGFβ1 for 18 hours increased the production of endogenous TGFβ1 and upregulated expression of TGFβ-R1, TGFβ-R2 and p-Smad3 (Figure 3A-B). These changes were accompanied by upregulation of γ-secretase proteinases including nicastrin, presenilin 1 and 2, PEN2 and Notch downstream effectors including Hes1 and Hes5 (Figure 3C-D). Similar to what we observed in Figure 2, RO4929097 significantly inhibited upregulation of proteins in both signaling pathways caused by TGFβ1 treatment. These results indicate that RO4929097 inhibits both signaling pathways in Müller cells after stimulation with TGFβ1.

### Notch and TGFβ ligands had additive effects on upregulation of ECM proteins in Müller cells *in vitro*

**Figure 4:**
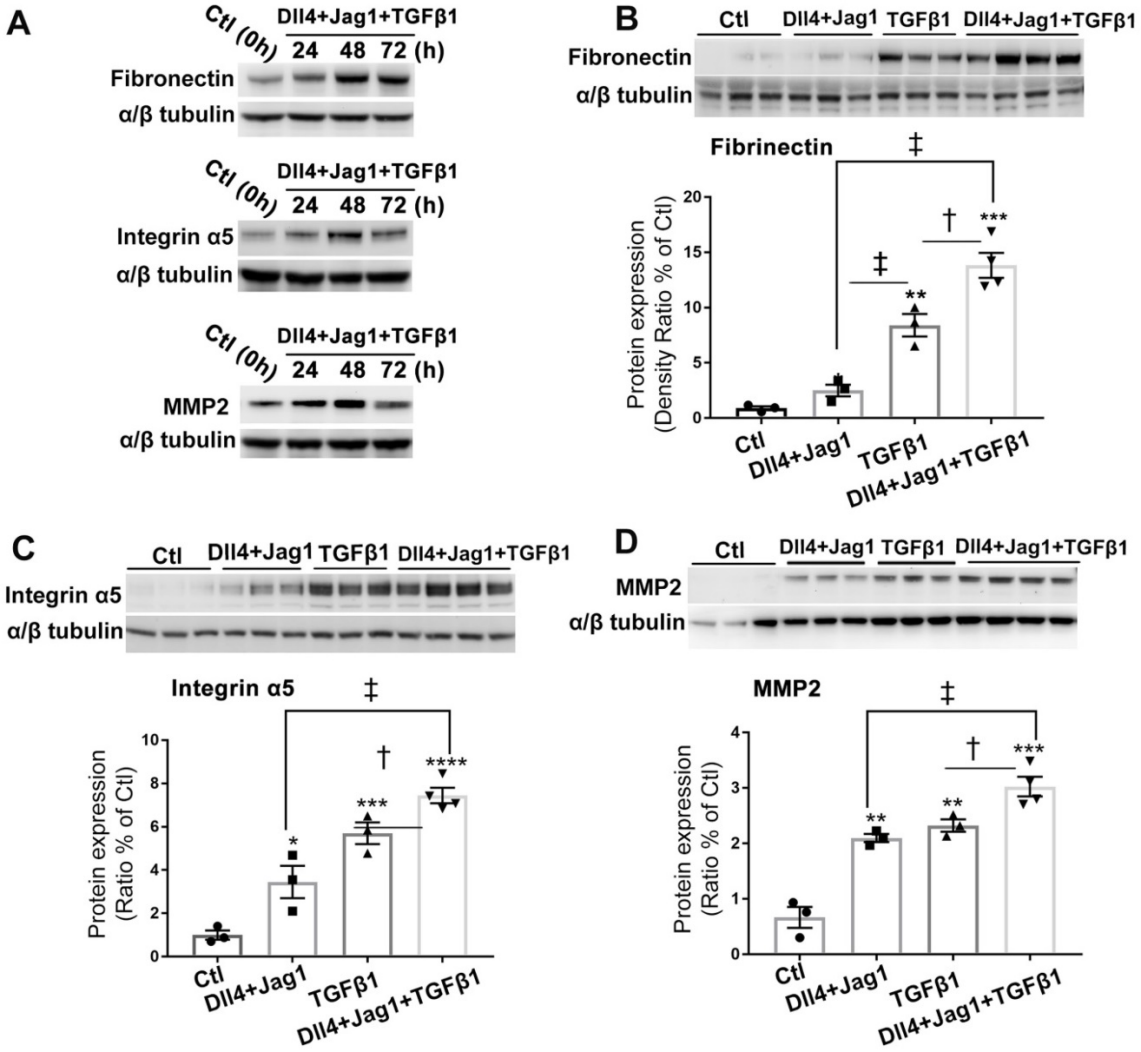
** Notch and TGFβ ligands have additive effects on upregulation of extracellular matrix (ECM) proteins in human Müller cells. (A)** Western blots for ECM proteins including fibronectin, intergrin α5 and matrix metallopeptidase 2 (MMP2) in Müller cells treated with Notch ligands Dll4 and Jagged 1 (Jag1, both 50 ng/ml) in combination with TGFβ1 (10 ng/ml) for 24, 48 or 72 hours. **(B-D)** Quantitative Western blot analyses of expression of fibronectin (B), intergrin α5 (C) and MMP2 (D) in Müller cells treated with Dll4 and Jagged1 (both 50 ng/ml), TGFβ1 (10 ng/ml) or a combination of both for 48 hours. A combination of Notch ligands and TGFβ showed additive effects on promoting overexpression of fibronectin, intergrin α5 and MMP2. *P<0.05, **P<0.01 and ***P<0.001, vs control (Ctl). ^†^P<0.05 and ^‡^P<0.01, compared between the 2 groups indicated. N=3/group. Independent repeats=2. Multiple comparison corrections were calculated using one-way ANOVA followed by Tukey's multiple comparison test, P < 0.0001 in** (B)**, P < 0.0001 in **(C)** , P < 0.0001 in **(D)**.

We next studied whether Notch and TGFβ ligands have additive effect on promoting Müller cells to overexpress ECM proteins. We optimised the duration of ligand treatment for upregulation of ECM proteins and found treatment of Müller cells with Dll4 and Jagged1 (both 50 μg/ml) in combination with TGFβ1 (10 ng/ml) for 48 hours strongly increased the expression of fibronectin, integrin α5 and matrix metalloproteinase-2 (MMP2) (Figure 4A). We then cultured Müller cells in media without or with Notch ligands including Dll4 and Jagged 1 (both 50 μg/ml), TGFβ1 (10 ng/ml) or a combination of both for 48 hours. We found that Notch ligands and TGFβ1 increased the expression of ECM proteins including fibronectin (Figure 4B), integrin α5 (Figure 4C) and MMP2 (Figure 4D), with the most profound effect observed with a combination of both Notch and TGFβ ligands (Figure 4B-D).

### RO4929097 inhibited overexpression of ECM proteins in Müller cells treated with Notch and TGFβ ligands

**Figure 5:**
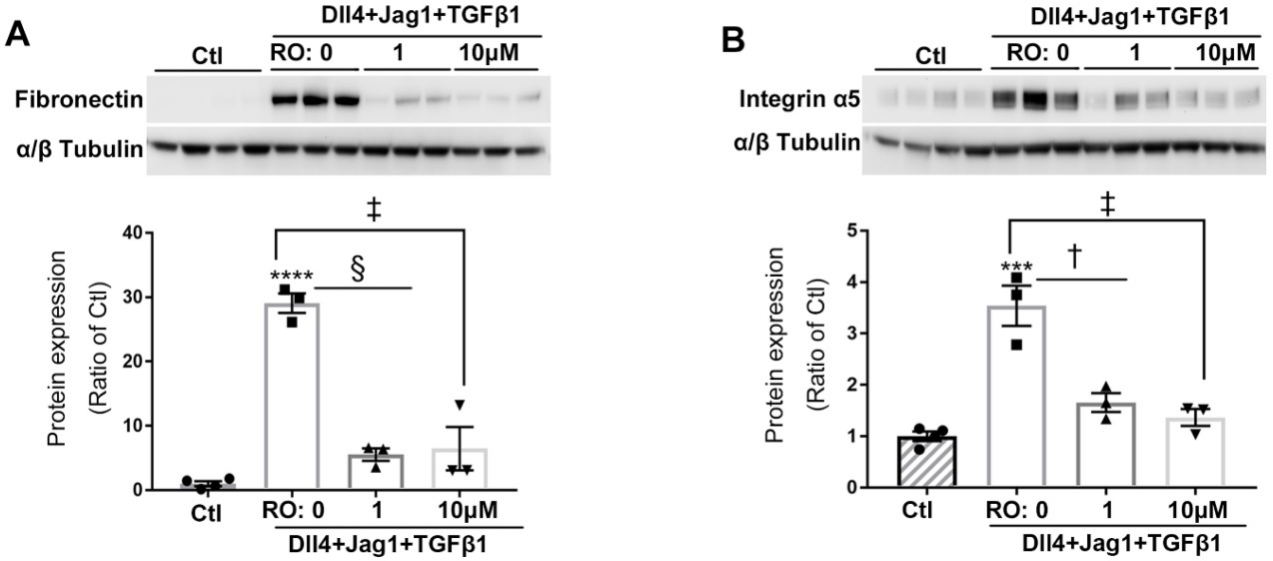
** Notch inhibition reduced expression of ECM proteins in human Müller cells treated with Notch and TGFβ ligands.** RO inhibited the overexpression of fibronectin **(A)** and integrin α5 **(B)** in Müller cells treated with Notch ligands Dll4 and Jagged1 (both 50 ng/ml) and TGFβ1 (10 ng/ml) for 24 hours. ****P<0.0001, ***P<0.001; vs Ctl. ^†^P<0.05, ^‡^P<0.01 and ^§^P<0.001, compared between the 2 groups indicated. N=3/group. Independent repeats=2. Multiple comparison corrections were calculated using one-way ANOVA followed by Tukey's multiple comparison test, P < 0.0001 in** (A)**, P < 0.0001 in **(B)**.

We next studied whether Notch inhibition using RO4929097 reduced the expression of ECM proteins after stimulation by ligands for both signaling pathways. Expression of fibronectin and integrin α5 was markedly increased in cells treated with Notch and TGFβ ligands and this effect was significantly inhibited by RO4929097 at a concentration as low as 1.0 μM (Figure 5).

**Figure 6:**
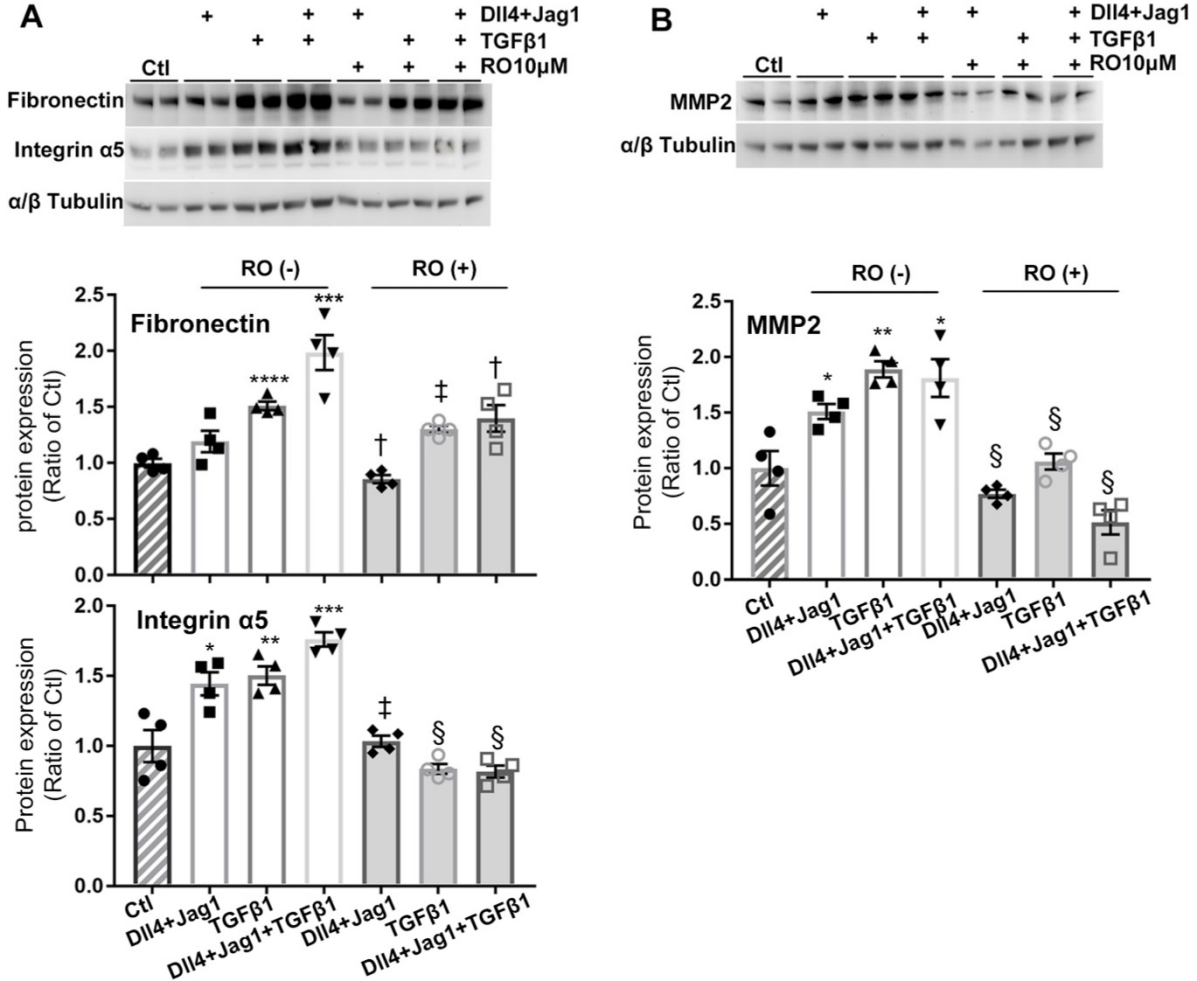
** RO4929097 (RO) inhibits overexpression of ECM proteins in human Müller cells treated with Notch and TGFβ ligands.** Treatment of Müller cells with Notch ligands (Dll4 and Jagged1, both 50 ng/ml) or TGFβ1 (10 ng/ml) for 24 hours promoted expression of fibronectin, intergrin α5 **(A)** and MMP2 **(B)**, with the most profound effects observed in the group treated with Notch ligands in combination with TGFβ1. RO treatment significantly inhibited overexpression of fibronectin, intergrin α5 and MMP2 in Müller cells stimulated by Notch ligands, TGFβ1 or a combination of both. *P<0.05, **P<0.01 and ***P<0.001, vs control (Ctl). ^†^P<0.05, ^‡^P<0.01 and ^§^P<0.001, compared with the corresponding group without RO treatment. N=4/group. Independent repeats=2. Multiple comparison corrections were calculated using one-way ANOVA followed by Tukey's multiple comparison test, P < 0.0001 for Fibronectin and P < 0.0001 for Integrin α5 in** (A)**, P < 0.0001 in **(B)**.

We further studied the details of RO4929097 on inhibiting ECM proteins in Müller cells stimulated with Notch ligands, TGFβ1 or both (Figure 6). We confirmed that Notch ligands and TGFβ1 had additive effects on promoting Müller cells to overexpress fibronectin, integrin α5 and MMP2 and these effects were inhibited by RO4929097 alone (Figure 6).

### RO4929097 inhibited the metabolic activity of Müller cells stimulated by Notch and TGFβ ligands but without damaging Müller cells

**Figure 7:**
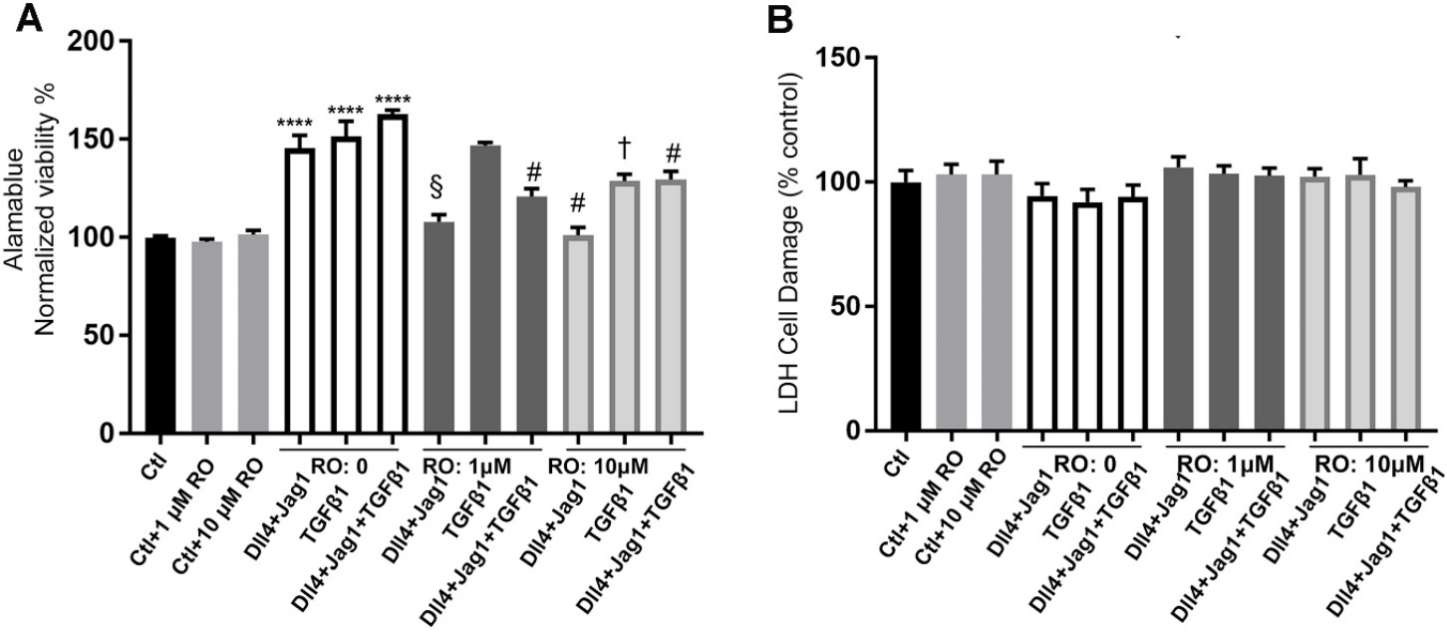
** The effects of Notch inhibition on Müller cell proliferation and cytotoxicity**. **(A)** AlamarBlue cell viability assays in Müller cells 24 hours after treatment with different ligands, with or without RO4929097 (RO). ****P<0.0001, vs control (Ctl, without RO); ^†^P<0.05, ^‡^P<0.01, ^§^P<0.001 and ^#^P<0.0001, vs corresponding groups treated with Notch ligands respectively; n=8/group, error bars = SEM. **(B)** LDH cytotoxicity assays in Müller cells 24 hours after treatment with the indicated strategies. There was no statistically significant difference among the treatment groups. N=8/group, error bars=SEM. Independent repeats=2. Multiple comparison corrections were calculated using one-way ANOVA followed by Tukey's multiple comparison test, P < 0.0001 in** (A)**, P = 0.4 in **(B)**.

The AlamarBlue assay was used to assess metabolic activity and the LDH assay to measure cytotoxicity of MIO-M1 cells after exposing them to different media and treatments for 24 hours. Treatment with the Notch ligands or TGFβ ligands, both alone and in combination, significantly increased the metabolic activity of MIO-M1 cells. RO inhibited the increased metabolic activity of MIO-M1 cells at concentrations of both 1 μM and 10 μM (Figure 7A) with no significant cytotoxic effects (Figure 7B).

### Chemically-induced retinal fibrosis in mice

**Figure 8:**
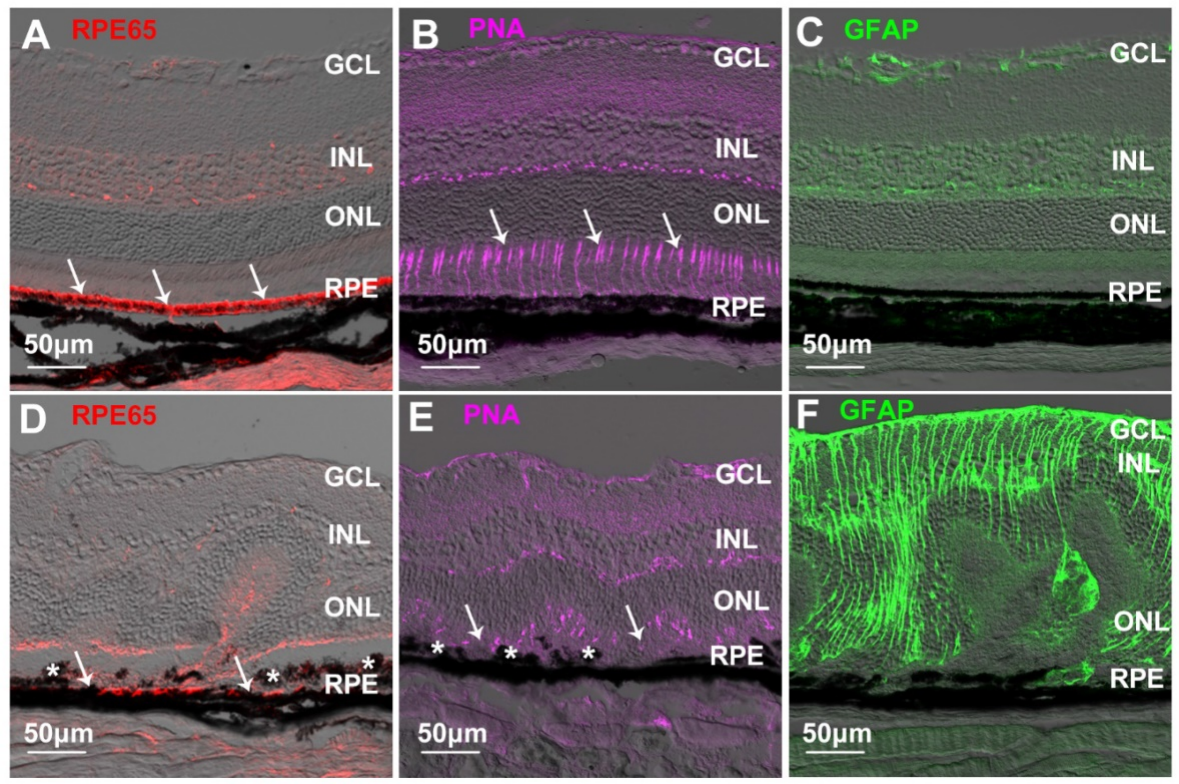
** RPE damage, photoreceptor degeneration and Müller cell gliosis in a mouse model of NaIO_3_-induced retinal damage. (A-C)** Immunostaining for RPE65 (A, arrows), peanut-agglutinin (PNA, arrows in B) and GFAP (C) in the normal retina. **(D-F)** Reduced immunoreactivity for RPE65 (D, arrows), loss of PNA-stained photoreceptor apical process (E, arrows) and Müller cell gliosis (F) after NaIO_3_-induced retinal injury. The asterisks in D and E indicate clumped RPE cells in retinas with NaIO_3_-induced injury. GCL=ganglion cell layer, INL=inner nuclear layer, ONL=outer nuclear layer, RPE=retinal pigment epithelium.

We next performed an intraperitoneal injection of NaIO_3_ to cause retinal fibrosis secondary to RPE damage and photoreceptor degeneration in mice 51. Normal RPE cells strongly expressed RPE65 (Figure 8A) along with a regular alignment of photoreceptor apical processes (Figure 8B). GFAP was confined to the superficial and the outer plexiform layers in the normal retina (Figure 8C). A single dose of intraperitoneal injection of NaIO_3_ (50 mg/kg) severely damaged the RPE, as shown by reduced expression of RPE65 and clumping of RPE cells seen on immunohistochemical studies (Figure 8D) which was accompanied by severe photoreceptor degeneration (Figure 8E) and Müller cell gliosis (Figure 8F). Double label IHC for CRALBP and GFAP was used to detect Müller cells in normal and NaIO_3_-damaged retinas (Figure S1). Immunoreactivity for CRALBP in gliotic Müller cells after NaIO_3_-induced retinal injury was reduced (Figure S1B) compared with the normal retina (Figure S1A).

### Intravitreal injection of RO4929097 inhibited Notch and TGFβ Signaling in retinas damaged by NaIO_3_

**Figure 9:**
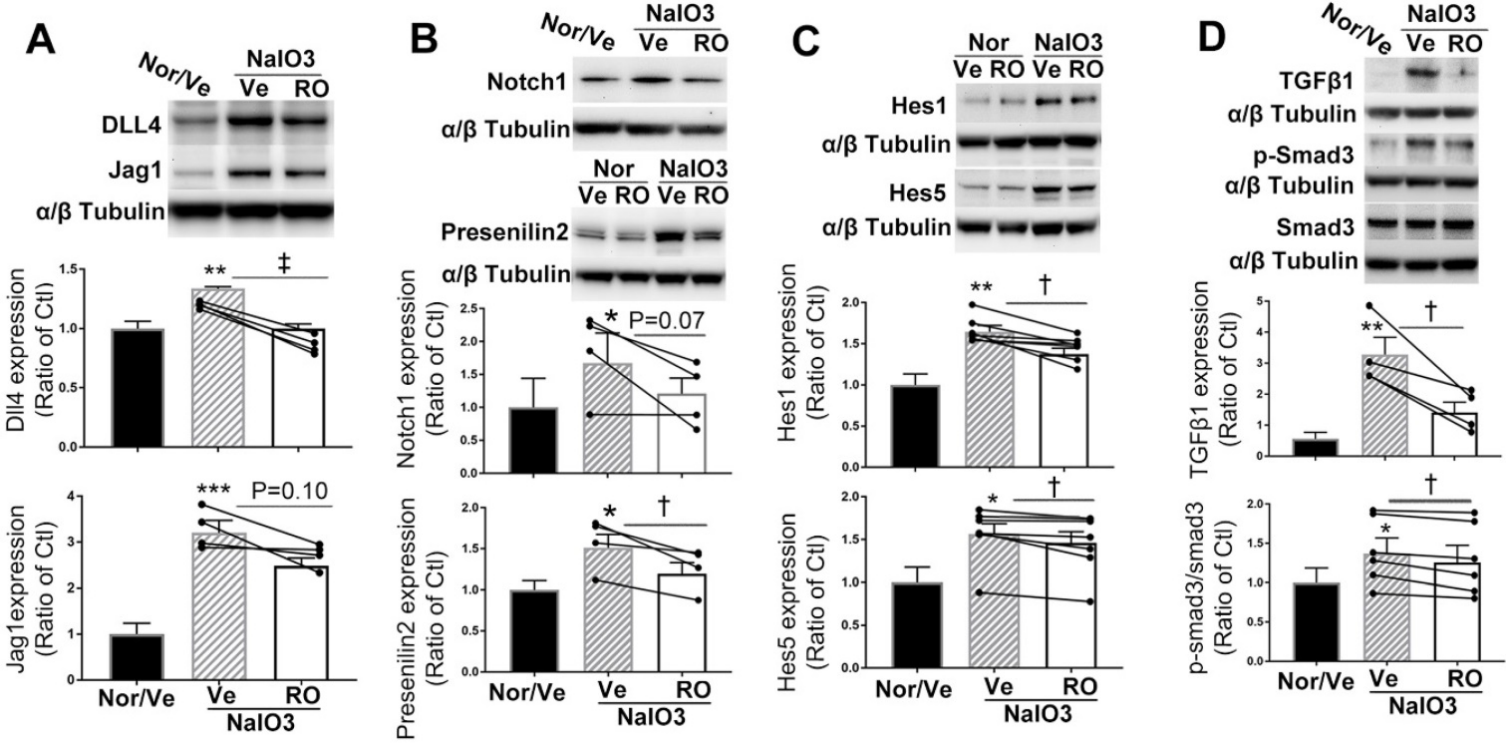
** Intravitreal injection of RO4929097 (RO) inhibits Notch and TGFβ signalling in mice with NaIO_3_-induced retinal damage.** Mice received intravitreal injections of RO or vehicle (Ve) immediately after NaIO_3_-induced retinal damage and data analyses were conducted 5 days later. **(A-D)** Western blot analyses of Notch signalling proteins including Notch ligands Dll4 and Jagged1 **(A)**, Notch receptor 1 and γ-secretase presenilin 2 **(B)**, Notch downstream effectors including Hes1 and Hes5 **(C)** as well as TGFβ signalling proteins including TGFβ1 and p-Smad3 **(D)**. *P<0.05, **P<0.01 and ***P<0.001, vs normal mice receiving vehicle (Nor/Ve). ^†^P<0.05, and ^‡^P<0.01, paired t-tests between RO and vehicle treated groups. N=4-7/group. Multiple comparison corrections were calculated using one-way ANOVA followed by Geisser-Greenhouse correction, P = 0.005 for Dll4 and P = 0.01 for Jagged1 in **(A)**, P = 0.04 for Notch1 and P = 0.006 for Presenilin2 in **(B)**, P = 0.002 for Hes1 and P = 0.01 for Hes5 in **(C)**, P = 0.0025 for TGFβ1 and P = 0.04 for p-Smad3 in **(D)**. Nor/Vehicle, normal eye receiving vehicle injection.

We performed an intravitreal injection of 1.0 µl of 100 μM RO4929097 to study whether it inhibits Notch and TGFβ signaling in retinas with NaIO_3_-induced fibrosis (Figure 9). Western blot analysis indicated that NaIO_3_-induced retinal damage was associated with significant upregulation of Notch signaling proteins, including Notch ligands Dll4 and Jagged1 (Figure 9A), Notch1, presenilin 2 (Figure 9B) and Notch downstream effectors including Hes1 and Hes5 (Figure 9C), with overexpression of TGFβ1 and p-Smad3 (Figure 9D), all of which were significantly inhibited by an intravitreal injection of RO4929097 (Figure 9A-D). These results indicate that the Notch and TGFβ signaling pathways are both activated by NaIO_3_-induced retinal damage and intravitreal injection of RO4929097 inhibits activation of both pathways.

### Intravitreal injection of RO4929097 prevented retinal gliosis resulting from NaIO_3_-induced retinal damage

**Figure 10:**
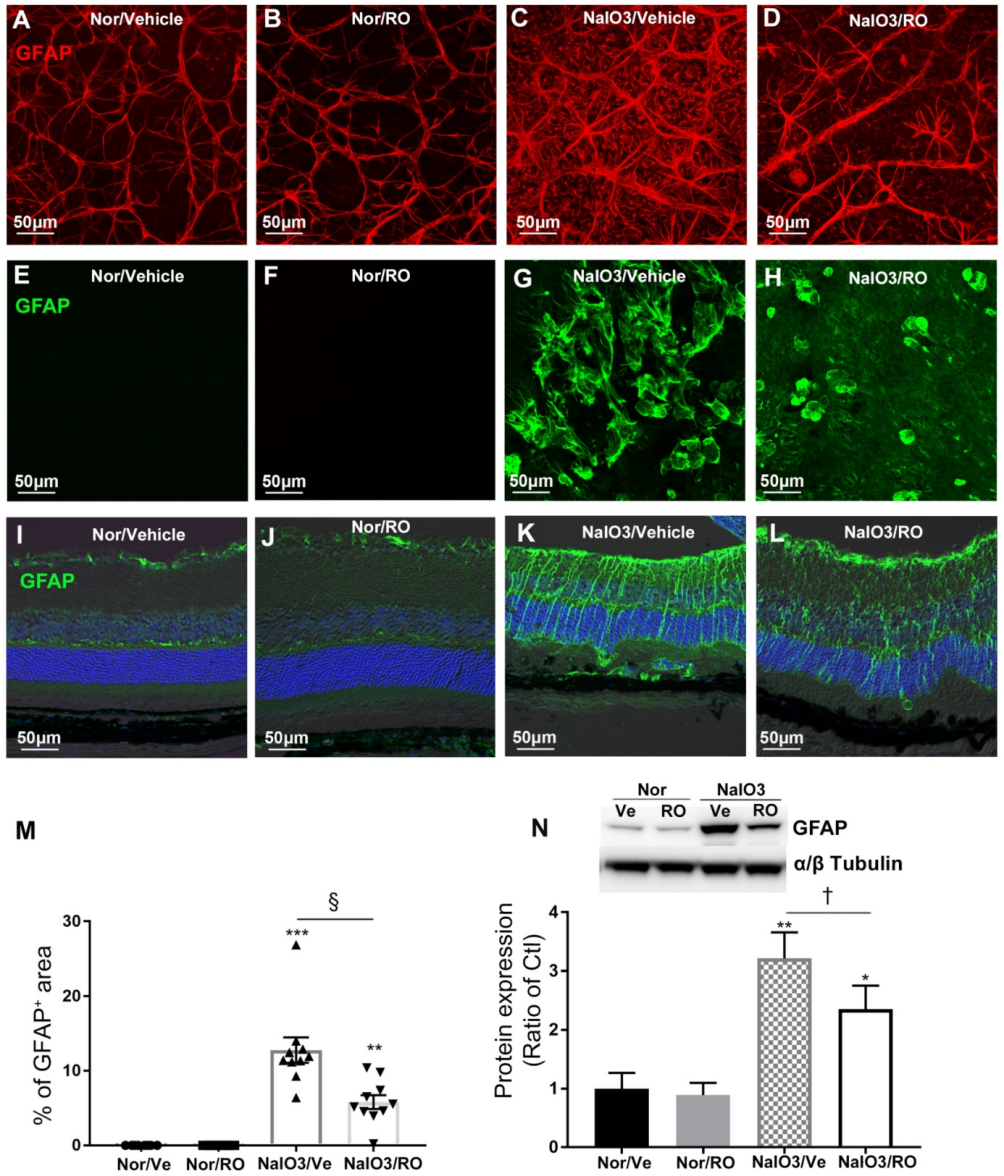
** Intravitreal injection of RO4929097 prevents Müller cell-dominated retinal fibrosis resulting from NaIO_3_-induced retinal damage.** Intravitreal injections were performed immediately after NaIO_3_-induced retinal damage (50 mg/Kg, intraperitoneal injection), with one eye receiving RO4929097 (RO, 1 μl/eye, 100 μM) and the contralateral eye injected with vehicle (Ve) in each mouse. Data analyses were conducted 5 days after treatment. **(A-D)** Immunostaining for glial fibrillary acidic protein (GFAP) on retinal flatmounts with the ganglion cell layer facing up. **(E-H)** Immunostaining for GFAP on retinal flatmounts with photoreceptors facing up. **(M)** Quantitative analyses of GFAP-strained areas on retinal flatmounts. **(I-L)** Immunostaining for GFAP on frozen sections. **(A, B, E, F, I and J)** normal (Nor) eyes receiving vehicle (A, E and I) or RO (B, F and J). **(C, D, G, H, K and L)** NaIO_3_-damaged eyes receiving intravitreal injections of vehicle (C, G and K) or RO (D, H and L). **(N)** Western blot analysis of GFAP expression using retinal proteins. **P<0.01 and ***P<0.001, vs normal mice receiving vehicle. ^†^P<0.05, and ^§^P<0.001, paired t-tests between RO and Ve treated groups. N=7-10/group. Multiple comparison corrections were calculated using one-way ANOVA followed by Tukey's multiple comparison test, P < 0.0001 in** (M)**, P = 0.0023 in **(N)**. Nor/Vehicle, normal eye receiving vehicle injection.

We conducted IHC and Western blots to study the effect of intravitreal injection of RO4929097 on upregulation of GFAP in NaIO_3_-induced retinopathy (Figure 10). RO4929097 did not obviously change the level or extent of GFAP immunoreactivity in the normal retina (Figure 10A-B, Figure 10E-F and Figure 10I-J). NaIO_3_-induced retinal damage led to marked Müller cell gliosis across the retina which was reduced by RO4929097 particularly in the superficial and the outer retina (Figure 10C-D, Figure 10G-H and Figure 10K-L). Our collective results from IHC and Western blots indicate that NaIO_3_-induced retinal damage upregulated GFAP which was significantly inhibited by intravitreal injection of RO4929097 (Figure 10M-N).

### Intravitreal injection of RO4929097 inhibited overexpression of ECM proteins and myofibroblast formation in the subretinal space after NaIO_3_-induced retinal damage

**Figure 11:**
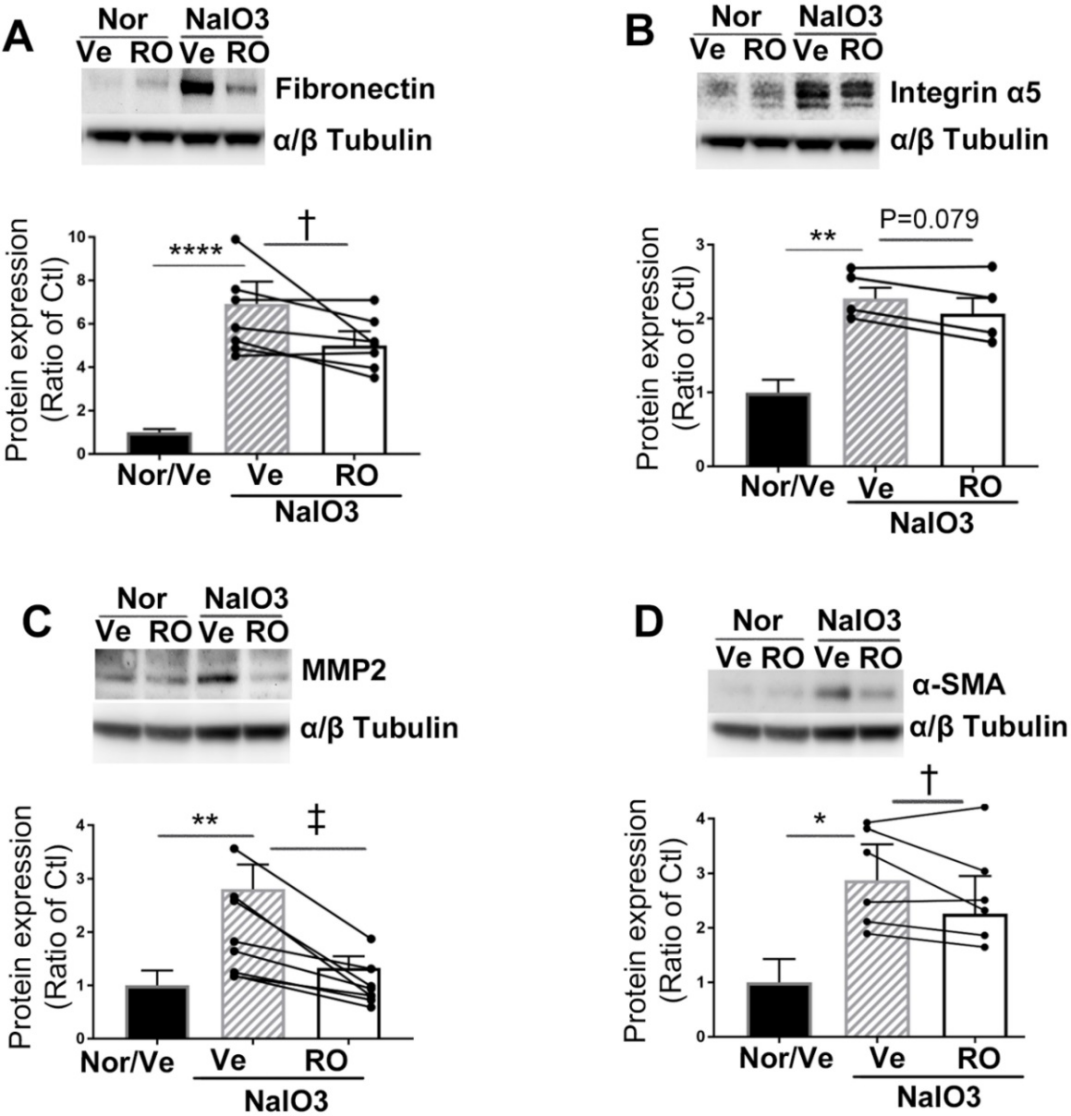
** Intravitreal injection of RO4929097 inhibits overexpression of ECM proteins resulting from chemically-induced retinal damage. (A-E):** Western blot analyses of ECM proteins including fibronectin **(A)**, integrin α5 **(B)**, MMP2 **(C)** and α-SMA**(D)** using retinal proteins. **P<0.01 and ****P<0.0001, vs normal mice receiving vehicle. ^†^P<0.05, and ^‡^P<0.01, paired t-tests between RO and Ve treated groups. N=4-7/group. Multiple comparison corrections were calculated using one-way ANOVA followed by Tukey's multiple comparison test, P < 0.0001 in **(A)**, P = 0.002 in **(B)**, P = 0.002 in **(C)**, P = 0.004 in **(D)**. Nor/Vehicle, normal eye receiving vehicle injection.

We performed Western blots to study the effects of intravitreal injection of RO4929097 on overexpression of ECM proteins (Figure 11). RO4929097 in normal eyes did not affect the levels of expression of ECM proteins, including fibronectin (Figure 11A), integrin a5 (Figure 11B), MMP2 (Figure 11C) and α-SMA (Figure 11D). However, NaIO_3_-induced retinal damage led to upregulation of all four ECM proteins which were significantly inhibited by intravitreal injection of RO4929097 (Figure 11A-D).

We performed double label IHC to study the transformation of myofibroblasts after NaIO_3_-induced retinal injury (Figure S2). NaIO_3_-induced retinal damage resulted in Müller cell gliosis and cell infiltration in the subretinal space, both of which were inhibited by intravitreal injection of RO4929097 (Figure S2A-D). Double labeling for GFAP and α-SMA indicated that some GFAP^+^ cells in the subretinal space were also positive for α-SMA, indicating their Müller cell origin (Figure S2E-H, arrows). Double labeling for α-SMA and Iba1 indicated that some Iba1^+^ cells in the subretinal space were also positive for α-SMA (Figure S2I-L, arrows), suggesting that some microglia/macrophages may have differentiated into myofibroblasts. We also found that disrupted RPE cells expressed α-SMA (Figure S2M-P, arrows), suggesting EMT of some surviving RPE. Our findings suggest that activated Müller cells, microglia/macrophages and disrupted RPE cells can potentially differentiate into myofibroblast-like cells to contribute to subretinal fibrosis.

### Intravitreal injection of RO4929097 reduces photoreceptor degeneration and microglial infiltration in the outer retina

We also performed double labelling for Iba1 and PNA in retinal flatmounts to study the effects of Notch inhibition using RO4929097 on microglial infiltration and photoreceptor degeneration in the *in vivo* model. NaIO_3_ induced extensive photoreceptor degeneration and microglial infiltration in the outer retina and both were significantly inhibited by intravitreal injection of RO4929097 (Figure S3).

### Intravitreal injection of RO4929097 did not affect the integrity of retinal vasculature and photoreceptor health in the normal retina

**Figure 12:**
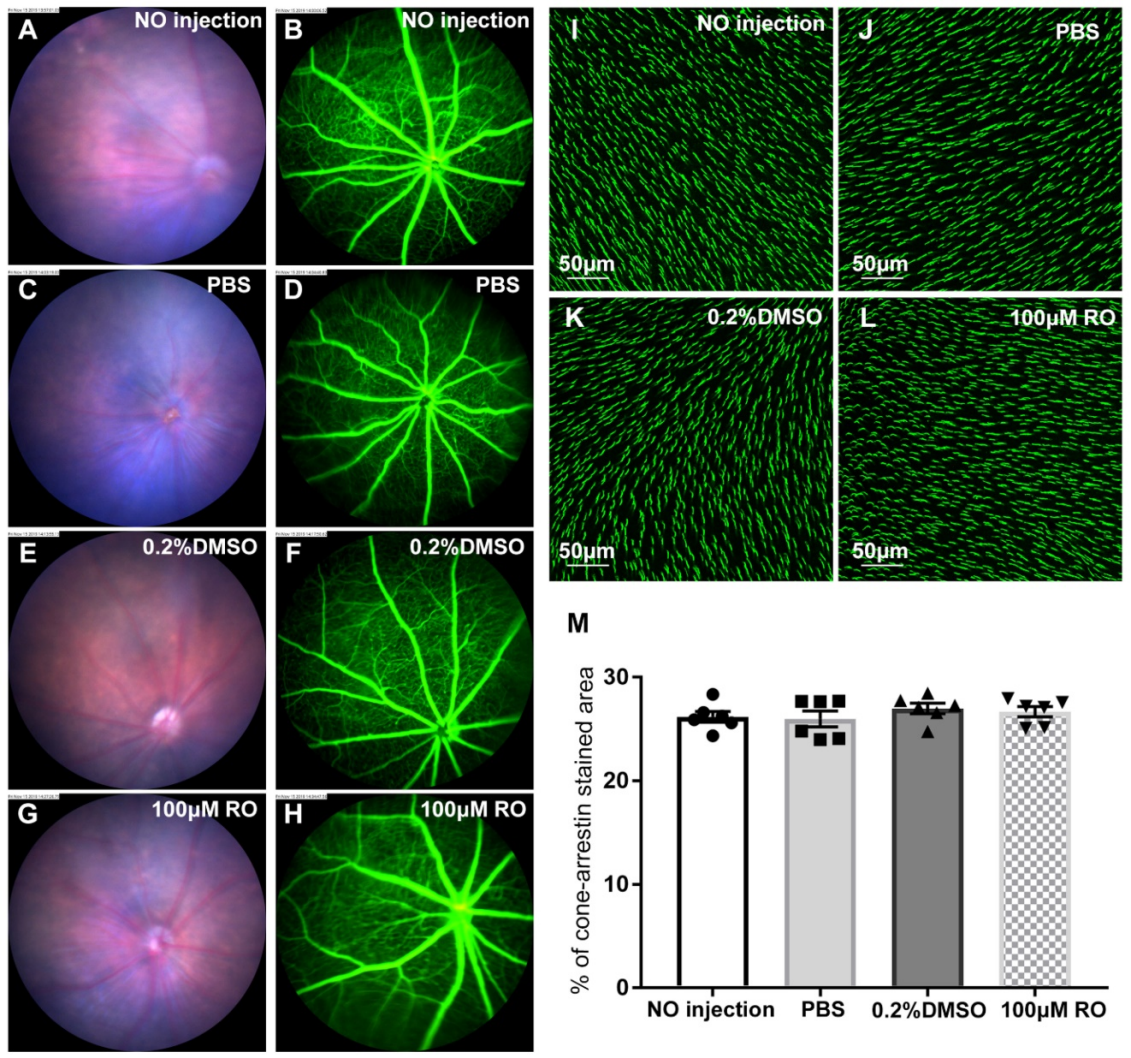
** The safety of intravitreal injection of RO4929097 in the normal retina. (A-H)** Color fundus photography (A, C, E and G) and fluorescein angiography (B, D, F and H) one week after intravitreal injection of 1.0 μl of 100 μM RO4929097, PBS, PBS containing 0.2% DMSO or no injection. **(I-L)** Representative images of retinal wholemounts stained with cone arrestin 17 days after intravitreal injection of test compounds. **(M)** Quantitative analyses of cone arrestin-stained areas in retinal wholemounts showed no damage to photoreceptors 17 days after injection of test compounds. N=6/group. Multiple comparison correction was calculated using one-way ANOVA followed by Tukey's multiple comparison test, P = 0.62 in (M).

We studied the safety of intravitreal injection of RO4929097 using fundus color photography, fluorescein angiography and immunostaining for cone arrestin, a marker of cone photoreceptor apical processes (Figure 12). We found that intravitreal injection of RO4929097 did not affect the fundus (Figure 12A, C, E and G), including the retinal vasculature (Figure 12B, D, F and H). Quantitative analyses of cone arrestin-stained retinal wholemounts indicated that RO4929097 did not damage cone photoreceptor apical processes when assessed 17 days after intravitreal injection of RO4929097 (Figure 12I-M).

## Discussion

Gliotic Müller cells develop fibroblast-like features as a major part of retinal fibrosis in conditions such as PVR, PDR, nAMD and IRDs 1-3, 5, 7, 8, 52. Here, we have studied whether the Notch and TGFβ signaling pathways are involved in the development of Müller cell-dominated retinal fibrosis *in vitro* and *in vivo*. Treatment of human MIO-M1 Müller cells with Notch ligands or TGFβ1 each activated both signaling pathways, resulting in overexpression of ECM proteins. Both DAPT and RO4929097 are selective γ-secretase protease inhibitors. As a recent study indicates that the potency of RO4929097 in inhibiting Notch signaling is 30 times stronger than DAPT 46, here we used RO4929097 to study the effect of Notch inhibition on retinal fibrosis *in vitro* and *in vivo*. We found that treatment of Müller cells with RO4929097 inhibited both Notch and TGFβ signaling pathways, leading to reduced expression of ECM proteins resulted from stimulation with ligand proteins from either signaling pathway or a combination of both. The Notch and TGFβ signaling pathways were activated in a mouse model of retinal fibrosis induced by intraperitoneal injection of NaIO_3_. Intravitreal injection of RO4929097 inhibited both signaling pathways, reduced overexpression of ECM proteins and prevented retinal fibrosis in this murine model. We found no concerns on the safety of RO4929097 after one dose injection and following up the injected normal mice for 17 days. Our collective findings indicate that the Notch and TGFβ signaling pathways contribute to retinal fibrosis and that inhibiting Notch signaling might be a way to prevent it. This study is of clinical significance in developing a treatment for preventing retinal fibrosis in humans.

We studied how the Notch and TGFβ signaling pathways contribute to fibrosis in Müller cells after treatment with ligand proteins from each pathway. We first identified that treatment of Müller cells with Notch or TGFβ ligands for 18 or 24 hours activated each signaling pathway profoundly. Subsequent experiments based on these durations of treatment found that stimulating Müller cells with Notch ligands, including Dll4 and Jagged1, induced upregulation of γ-secretase proteases and Notch downstream effectors, including Hes1 and Hes5, and this effect was accompanied by upregulation of endogenous TGFβ1, TGFβ receptors and p-Smad3, indicating that Notch ligand treatment activated both signaling pathways. Similarly, treatment of Müller cells with TGFβ1 upregulated TGFβ receptors and p-Smad3, with upregulation of Notch signaling proteins, suggesting that TGFβ1 treatment also activated both signaling pathways. Importantly, we found that Notch and TGFβ signaling had additive effects on promoting Müller cell to overexpress ECM proteins and such effects were inhibited by the γ-secretase inhibitor RO4929097. Our results are consistent with previous observations that Notch and TGF-β signaling pathways function collaboratively to contribute to fibrosis 53. In liver fibrosis, TGFβ1 upregulates p-Smad2/3, Jagged1, Notch1 and Hes1, leading to trans-differentiation of hepatic stellate cells into myofibroblast cells 54. In kidney fibrosis, TGFβ1 induces EMT of kidney tubular cells through upregulation of Jagged1 and Hes1 55. Notch inhibition ameliorates renal fibrosis through inhibiting TGFβ/Smad2/3 signaling 56. All these findings formed a foundation for our hypothesis that targeting Notch signaling is a way to prevent retinal fibrosis.

TGFβ signaling has been reported to induce EMT and overexpression of ECM proteins in RPE cells through upregulation of Notch signaling proteins 29, 57. In PVR, TGFβ isoforms regulate the synthesis and degradation of ECM proteins both *in vitro* and *in vivo*, leading to collagen accumulation and fibrosis 58. Previous IHC studies have identified the presence of Müller cells in epiretinal fibrotic tissues associated with PVR and PDR 2, 5, 6. Here we found that Müller cells have the capacity to produce ECM proteins including fibronectin, integrin α5 and MMP2, thus providing additional information on the contribution of Müller cells to retinal fibrosis.

We used a mouse model of Müller cell gliosis caused by intraperitoneal NaIO_3_ to study changes in Notch and TGFβ signaling and tested the effects of intravitreal injection of RO4929097 on Müller cell gliosis. NaIO_3_ is a chemical oxidizing agent that was reported to primarily damage the RPE, leading to subsequent photoreceptor degeneration and alterations in retinal structures including Müller cell gliosis 51, 59-61. Müller cell gliosis has been found on the surface of the retina and in the subretinal space in a previous study using the NaIO_3_-induced retinal damage model in rabbits 61. Our IHC studies indicated that similar changes were observed after a single dose of NaIO_3_ injection (50 mg/kg) in mice. We also found that NaIO_3_-induced retinal damage was accompanied by upregulation of Notch and TGFβ signaling proteins. Importantly, both signaling pathways could be effectively inhibited by intravitreal injection of RO4929097.

We performed IHC and Western blots to study the effects of Notch inhibition on the development of preretinal and subretinal fibrosis. Results from immunostaining for GFAP on retinal wholemounts and frozen sections indicate that intravitreal injection of RO4929097 prevented Müller cell gliosis on the surface and in the subretinal space. Western blots confirmed that Notch inhibition significantly reduced overexpression of GFAP and ECM proteins, including fibronectin, integrin α5, MMP2 and α-SMA. Our in vivo studies using flatmount retinas and frozen sections suggested that Müller cells became gliotic to contribute to preretinal (Figure 10A-D), intraretinal and subretinal fibrosis (Figure 10E-L). Results from double label IHC on frozen sections indicate that activated Müller cells, microglia/macrophages and disrupted RPE cells can potentially differentiate into myofibroblast-like cells to contribute to subretinal fibrosis (Figure S2). Our results are consistent with previous reports that Müller cells, microglia/macrophages and RPE cells are the major sources of retinal fibrosis in PVR, PDR and nAMD 3, 62-64.

Müller cells are a rich source of cytokines and inflammatory factors that may induce cell migration and proliferation, thus contributing to retinal fibrosis 2. We have studied the effect of Notch inhibition on infiltration of activated microglia and photoreceptor degeneration in the in vivo model. We found that Notch inhibition by RO4929097 reduced the number of Iba1^+^ cells in the outer retina and that this effect was accompanied by attenuation of photoreceptor degeneration (Figure S3). The attenuated photoreceptor degeneration might be attributed to reduced retinal gliosis and fewer microglia infiltrating into the outer retina after Notch inhibition. Future research is warranted to study the effect of intravitreal injection of RO4929097 on subretinal fibrosis, microglial activation, EMT of RPE cells, photoreceptor degeneration and expression cytokines and chemokines in multiple animal models such as JR5558 mice 65-67 and laser-induced subretinal fibrosis and neovascularization 68, 69.

There are several limitations of this study. Previous studies indicate that p-Smad3 may directly interact with cleaved NICD to promote transcription of Hes1 in myogenic cells 53 and that TGFβ1 induces EMT through upregulating Hes1 and Jagged1 in the RPE 55. Our *in vitro* data indicate that the Notch and TGFβ signaling pathways both contributed to overexpression of ECM proteins and that this effect could be inhibited by treatment with RO4929097. Notch inhibition by RO4929097 also downregulated endogenous TGFβ1, TGFβ receptors and phosphorylation of Smad3 in Müller cells. However, we have not identified the point of convergence of the two pathways, nor have we established whether selective inhibition of TGFβ signaling affects the Notch signaling pathway. Further research is warranted to study whether selective inhibition of TGFβ signaling using an agent such as SB431542 50, 54 affects expression of Notch ligands, receptors, γ-secretase proteases and Notch downstream effector proteins in Müller cells stimulated by recombinant TGFβ1.

Another limitation of this study is that NaIO_3_-induced Müller cell gliosis may not recapitulate all the features of retinal fibrosis in human diseases such as nAMD. Müller cell gliosis is a secondary response to photoreceptor degeneration due to widespread necrosis of the RPE in this model 70-72. There is no subretinal neovascularization and less involvement of transdifferentiated RPE cells. Further studies are warranted to test whether intravitreal RO4929097 inhibits EMT of RPE cells and prevents subretinal fibrosis in other models such as JR5558 mutant mice 65, 66 and laser-induced subretinal fibro-neovascularisation 68, 69.

## Conclusions

In summary, our study indicates that both the Notch and TGFβ signaling pathways contribute to retinal fibrosis. Activation of Notch and TGFβ signaling pathways had additive effects on promoting Müller cells to overexpress ECM proteins and such effects could be inhibited by RO4929097. Intravitreal RO4929097 prevented preretinal and subretinal fibrosis resulting from NaIO_3_-induced retinal damage *in vivo*. This study is of clinical significance for the development of a new treatment to prevent retinal fibrosis in PVR, PDR and nAMD.

## Supplementary Material

Supplementary figures.

## Figures and Tables

**Table 1:** Antibodies used for Western blots

Antibody	Source and Cat#	Host	Dilution
Dll4	Abcam#7280	Rabbit	1:1000
Fibronectin	Chemicon#AB2033	Rabbit	1:1000
GFAP	Cell Signaling#3670	Mouse	1:1000
Hes1	Abcam#119776	Mouse	1:1000
Hes5	Abcam#25374	Rabbit	1:500
Integrin α5	Cell Signaling#98204	Rabbit	1:1000
Jagged1	Abcam#7771	Rabbit	1:1000
MMP2	Novusbio#AF1488-SP	Goat	1:500
Nicastrin	Cell Signaling#5665	Rabbit	1:1000
Notch1	Cell Signaling#3068	Rabbit	1:1000
PEN2	Cell Signaling#8598	Rabbit	1:1000
phos-smad3	Cell Signaling#9520	Rabbit	1:1000
phos-smad2/3	Cell Signaling#8828	Rabbit	1:1000
Presenilin 1	Cell Signaling#5643	Rabbit	1:1000
Presenilin 2	Cell Signaling#9979	Rabbit	1:1000
smad3	Cell Signaling#9523	Rabbit	1:1000
smad2/3	Cell Signaling#8685	Rabbit	1:1000
TGFβ-pan	Cell Signaling#3711	Rabbit	1:1000
α-SMA	Cell Signaling#48938	Rabbit	1:1000
β-actin	Cell Signaling#4967	Rabbit	1:2000
a/β tubulin	Cell Signaling#2148	Rabbit	1:2000

## Abbreviations

α-SMA: α-smooth muscle actin
Dll4: Delta-like 4
ECM: Extracellular matrix
GFAP: Glial fibrillary acidic protein
Hes1: hairy and enhancer of split-1
Iba1: ionized calcium binding adaptor molecule 1
LDH: lactate dehydrogenase
Jag1: Jagged1
MMP2: Matrix metalloproteinase-2
nAMD: Neovascular age-related macular degeneration
NaIO_3_: Sodium iodate
NICD: Notch intracellular domain
PBS: phosphate-buffered saline
PEN2: Presenilin enhancer 2
PFA: Paraformaldehyde
PNA: Peanut-agglutinin
p-Smad3: Phosphorylated Smad3
RO: RO4929097
RPE: retinal pigment epithelium cells
SEM: standard error of mean
TGFβ: Transforming growth factor β
VEGF: vascular endothelial growth factor
