# Supplementary material for: Targeting the Notch and TGF-β signaling pathways to prevent retinal fibrosis in vitro and in vivo
Source: Theranostics. 2020 Jun 29;10(18):7956–73. doi: 10.7150/thno.45192 (PMC7381727; doi:10.7150/thno.45192)
Supplement: Supplementary file 1 — Supplementary figures. [file thnov10p7956s1.pdf]

**Targeting the Notch and TGF-β signaling pathways to prevent retinal fibrosis *in vitro* and *in vivo***

Jiawen Fan[1, 2], Weiyong Shen[1*], So-Ra Lee[1], Ashish Easow Mathai[1], Rui Zhang[1], Gezhi Xu[2] and Mark C. Gillies[1*]

[1]The University of Sydney, Save Sight Institute, Discipline of Ophthalmology, Sydney Medical School, Sydney, New South Wales, Australia.

[2] Department of Ophthalmology and Vision Sciences and Key Laboratory of Myopia of State Health Ministry, Eye and ENT Hospital, Shanghai Medical College, Fudan University, Shanghai, People's Republic of China.

***Correspondence should be addressed to:**

Weiyong Shen

Save Sight Institute

The University of Sydney

Sydney 2000, Australia

E-mail: weiyongshen1@gmail.com

Mark C. Gillies

Save Sight Institute

The University of Sydney

Sydney 2000, Australia

E-mail: mark.gillies@sydney.edu.au

## Supplementary Figures

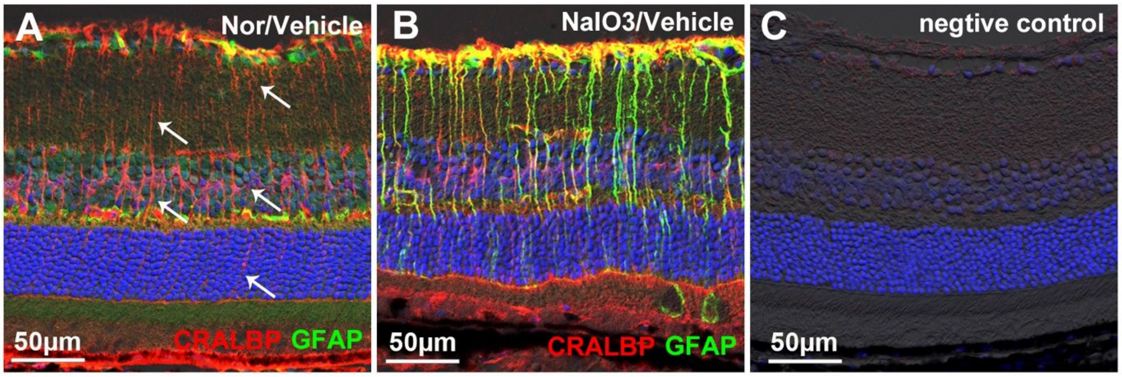


**Supplementary Figure 1. Phenotypic characterisation of Müller cells in normal and $NaIO_3$-damaged retinas. (A, B):** Double label IHC for CRALBP (red, arrows) and GFAP (green) showing reduced immunoreactivity for CRALBP in gliotic Müller cells after $NaIO_3$-induced retinal injury (B) compared with the normal retina (A). Nor/Vehicle, normal eye receiving vehicle injection. **(C):** Negative control without primary antibodies.

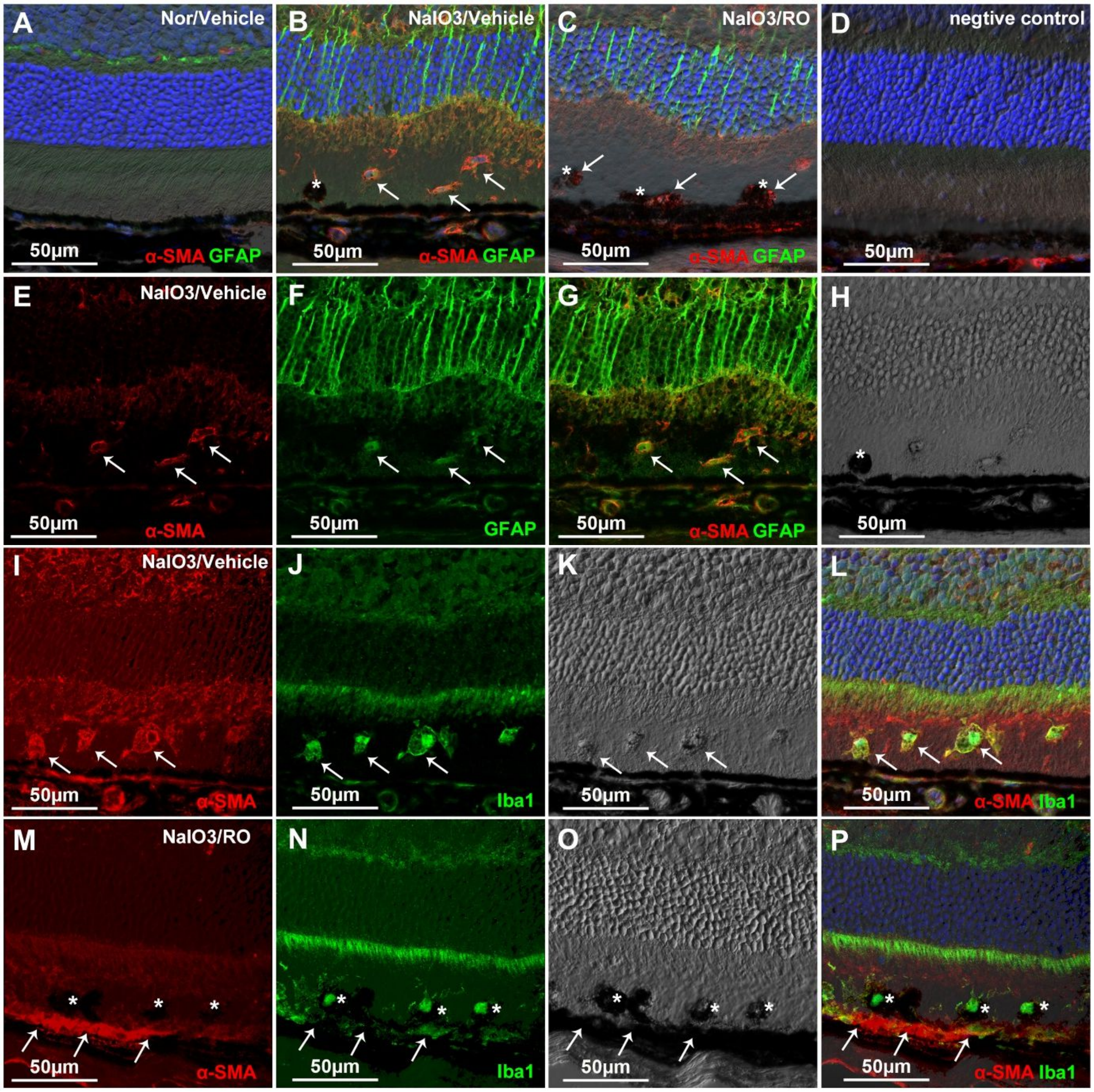


**Supplementary Figure 2. Myofibroblast formation in subretinal space after chemically-induced retinal damage. (A-C):** Double label IHC on retinal frozen sections using antibodies against α-SMA (a marker of myofibroblast, red) and GFAP (a marker of Müller cell gliosis, green). **(D):** Negative control without primary antibodies. **(E-H):** Separated staining of Fig. S1B to show cells double labelled for GFAP and α-SMA, indicating that some cells in the subretinal space may derive from Müller cells. **(I-P):** Double label IHC using antibodies against α-SMA (red) and Iba1 (a marker of microglia, green) in $NaIO_3$-damaged retinas. The pigment-laid cells (asterisks in B, C, G and O) may be attributed to necrotic RPE cells phagocytosed by macrophages. Nor/Vehicle, normal eye receiving vehicle injection.

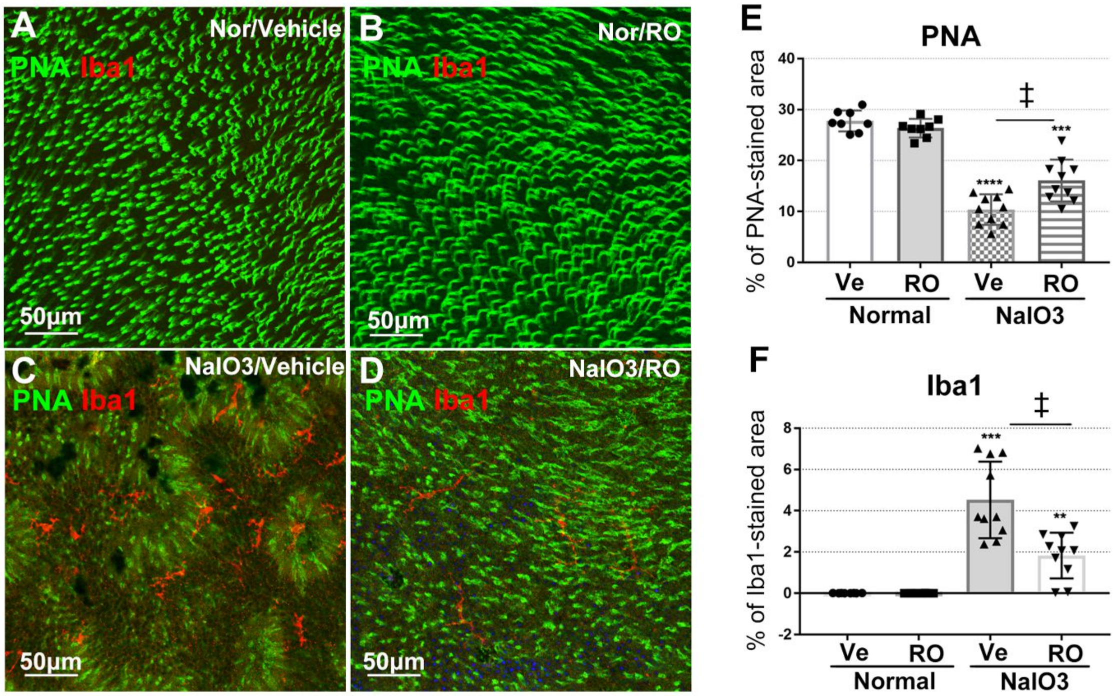


**Supplementary Figure 3. Intravitreal injection of RO4929097 reduces photoreceptor degeneration and microglial infiltration into the outer retina. (A-D):** Staining of retinal flatmounts using fluorescently labelled PNA (green) and an antibody against Iba1 (a marker of microglia, red). **(E** and **F)**: Quantitative analyses of PNA- and Iba1-stained areas in retinal flatmounts with photoreceptors facing up. ***P<0.001 and ****P<0.0001, vs normal mice receiving vehicle or RO. ‡P<0.01, paired t-tests between RO and Ve treated groups. N=10/group. Multiple comparison correction were calculated using one-way ANOVA followed by Tukey's multiple comparison test, P < 0.0001 in **(E)**, P = 0.002 in **(F)**. Nor/Vehicle, normal eye receiving vehicle injection.
